# Endothelial Lipase Concentrations Are Increased in Metabolic Syndrome and Associated with Coronary Atherosclerosis

**DOI:** 10.1371/journal.pmed.0030022

**Published:** 2005-12-20

**Authors:** Karen O Badellino, Megan L Wolfe, Muredach P Reilly, Daniel J Rader

**Affiliations:** **1**Schools of Nursing and Medicine, Institute for Translational Medicine and Therapeutics, University of Pennsylvania, Philadelphia, Pennsylvania, United States of America; Stanford UniversityUnited States of America

## Abstract

**Background:**

Endothelial lipase (EL), a new member of the lipase family, has been shown to modulate high-density lipoprotein (HDL-C) metabolism and atherosclerosis in mouse models. We hypothesized that EL concentrations would be associated with decreased HDL-C and increased atherosclerosis in humans.

**Methods and Findings:**

Healthy individuals with a family history of premature coronary heart disease (*n* = 858) were recruited as part of the Study of the Inherited Risk of Atherosclerosis. Blood was drawn in the fasting state before and, in a subgroup (*n* = 510), after administration of a single dose of intravenous heparin. Plasma lipids were measured enzymatically, lipoprotein subclasses were assessed by nuclear magnetic resonance, and coronary artery calcification (CAC) was quantified by electron beam computed tomography. Plasma EL mass was measured using a newly developed enzyme-linked immunosorbent assay. Median EL mass in pre-heparin plasma was 442 (interquartile range = 324–617) ng/ml. Median post-heparin mass was approximately 3-fold higher, 1,313 (888–1,927) ng/ml. The correlation between pre-heparin EL mass and post-heparin EL mass was 0.46 (*p* < 0.001). EL mass concentrations in both pre- and post-heparin plasma significantly correlated with all NCEP ATPIII-defined metabolic syndrome factors: waist circumference (*r* = 0.28 and 0.22, respectively, *p* < 0.001 for each), blood pressure (*r* = 0.18 and 0.24, *p* < 0.001 for each), triglycerides (*r* = 0.22, *p* < 0.001; and 0.13, *p* = 0.004), HDL cholesterol (*r* = –0.11, *p* = 0.002; and –0.18, *p* < 0.001), and fasting glucose (*r* = 0.11 and 0.16, *p* = 0.001 for both). EL mass in both routine (odds ratio [OR] = 1.67, *p* = 0.01) and post-heparin (OR = 2.42, *p* = 0.003) plasma was associated with CAC as determined by ordinal regression after adjustment for age, gender, waist circumference, vasoactive medications, hormone replacement therapy (women), and established cardiovascular risk factors.

**Conclusions:**

We report, to our knowledge for the first time, that human plasma EL concentrations, in both post-heparin and routine pre-heparin plasma, are significantly associated with metabolic syndrome features and with subclinical atherosclerosis. EL may be a pro-atherogenic factor in humans, especially in overweight individuals and those with metabolic syndrome.

## Abstract 2

Studies in rodents had suggested that endothelial lipase might play a role in the development of atherosclerosis. This study finds an association between EL levels and early-stage atherosclerosis in humans.

## Introduction

Two members of the lipase family, lipoprotein lipase (LPL) and hepatic lipase (HL) are known to influence plasma lipoprotein metabolism and risk of atherosclerosis in humans [1–5]. Endothelial lipase (EL) is a more recently discovered member of this family of lipases [6,7]. In contrast to LPL and HL, it is expressed in endothelial cells and, in the majority of reports, has relatively more phospholipase activity than the other two enzymes. Similar to HL and LPL, it is secreted and binds to the endothelial surface. In vitro, EL effectively hydrolyzes high-density lipoprotein (HDL) phospholipids. In mice, EL has a major influence on HDL metabolism. Adenovirally mediated overexpression of human EL in mice dramatically reduced HDL cholesterol (HDL-C) concentrations [6], shown to be due to rapid catabolism of HDL [8]. Transgenic overexpression of human EL was shown to reduce HDL-C concentrations as well [9]. Conversely, antibody inhibition of EL in mice significantly increased HDL-C concentrations [10], and EL knockout mice have a significant increase in HDL-C concentrations [9,11]. Furthermore, EL knockout mice crossed onto the apolipoprotein E knockout background have decreased atherosclerosis [12]. Based on these studies in mice, it has been suggested that EL may be an important risk factor and may affect the development of atherosclerosis in humans.

The relationship of EL to variation in lipoprotein concentrations and atherosclerosis in humans has not been reported. Several genetic association studies have suggested that rare variants and common polymorphisms in the human EL gene might be associated with variation in HDL-C concentrations [11,13,14], but they have not been conclusive and were not accompanied by direct measurement of EL concentrations. We hypothesized that concentrations of EL are inversely associated with HDL-C concentrations and directly associated with the metabolic syndrome and atherosclerosis in humans. To test this, we developed an immunoassay for human EL and used it to measure EL mass concentrations in both pre-heparin and post-heparin plasma samples in individuals enrolled in the Study of the Inherited Risk of Atherosclerosis (SIRCA). We determined the association of EL concentrations with lipoprotein concentrations, other cardiovascular risk factors and coronary artery calcification (CAC), a noninvasive measure of coronary atherosclerosis [15–17].

## Methods

### Study Protocol

SIRCA is a cross-sectional study of asymptomatic individuals and their families designed to investigate novel biomarkers and genetic factors associated with coronary atherosclerosis. The study design and initial findings have previously been published [18,19]. Briefly, persons were eligible for SIRCA if they had a family history of premature coronary artery disease (CAD), were free of clinical CAD, and were men, 20–75 years of age or women, 30–75 years. Exclusion criteria included other major CAD risk factors: known diabetes, total cholesterol higher than 300 mg/dl, cigarette smoking of one pack or more per day, or blood pressure higher than 160/100 mm Hg. The University of Pennsylvania Institutional Review Board approved the study protocol. Informed consent was obtained from each participant. This report focuses on 858 random unrelated individuals from the SIRCA in whom plasma EL concentrations were measured.

### Evaluated Parameters

Participants were assessed at the General Clinical Research Center in the University of Pennsylvania Medical Center after a 12-h overnight fast. A questionnaire was administered; height, weight, waist circumference, and blood pressure were obtained; and 30 ml of whole blood was drawn (“pre-heparin” plasma) into EDTA-containing tubes. In a subset of individuals (*n* = 510), blood was drawn 10 min after intravenous administration of heparin (60 units per kilogram body weight). After the blood was centrifuged, the plasma was removed and stored at –80 °C until use (“post-heparin” plasma). Plasma total cholesterol, HDL cholesterol, and triglyceride concentrations were measured enzymatically on a Cobas Fara II (Roche Diagnostic Systems, Indianapolis, Indiana, United States) using Sigma reagents (Sigma, St. Louis, Missouri, United States) in a Centers for Disease Control-standardized lipid laboratory. LDL cholesterol was calculated using the Friedewald formula.

Post-heparin plasma samples in EDTA were analyzed for lipoprotein subclasses by nuclear magnetic resonance (NMR) [20–22] (LipoSciences, Raleigh, North Carolina, United States). The NMR method uses the characteristic proton signals broadcast by lipoprotein subclasses to determine both the size and the quantity of each size of lipoprotein. A summary of the reporting format can be found at http://www.liposcience.com. The ten subclasses (and their size ranges) routinely used are: large very low-density lipoprotein (VLDL) (60–200 nm), intermediate VLDL (35–60 nm), small VLDL (27–35 nm), IDL (23–27 nm), large low-density lipoprotein (LDL) (21.3–23 nm), intermediate LDL (19.8–21.2 nm), small LDL (18.3–19.7 nm), large HDL (8.8–13.0 nm), intermediate HDL (8.2–8.8 nm), and small HDL (7.3–8.2 nm).

Electron beam computed tomography was performed on an Imatron C-150 XP/LXP Evolution scanner (Imatron, San Francisco, California, United States) connected to a MagicView workstation for volumetric image reconstruction. Using ECG triggering with the individual in a supine position, 40 contiguous, 3 mm-thick axial slices were acquired just below the carina through the apex of the heart. Global CAC scores were determined according to the method of Agatston [23].

### Sandwich Enzyme-Linked Immunosorbent Assay for Human EL

Detailed information on the development and quality control of the sandwich ELISA can be found in Protocol S1. The wells of a 96-well microtiter plate were coated with rabbit anti-human EL antibody. Various concentrations of purified recombinant human EL in phosphate-buffered saline containing 1% BSA were added to the wells as a standard. Plasma samples were diluted 1:10 in phosphate-buffered saline and applied to the wells. Specifically bound protein was incubated with biotin-conjugated rabbit anti-human EL antibody, followed by streptavidin-horseradish peroxidase conjugate, and detection with o-phenylenediamine. The reaction was stopped with 2.5 M sulfuric acid and the plate read at 490 nm. A standard curve of 490 nm absorbance versus the known concentrations of EL was constructed. The concentration of the plasma samples was determined by comparison to the standard curve multiplied by the dilution factor. Using this ELISA, EL mass concentrations were quantified in plasma collected in the routine manner (“pre-heparin” plasma) in the full sample (*n* = 858) as well as in the post-heparin plasma samples described above (*n* = 510).

### Statistical Analysis

Data are reported as median and interquartile range (IQR) or mean ± standard deviation for continuous variables and as proportions for categorical variables. The distributions of both pre-heparin and post-heparin EL mass concentrations were highly skewed rightward, so analyses were performed on log-transformed data. Variables were determined to be normally distributed using the Shapiro-Wilk test. Spearman correlations of EL mass with cardiovascular risk factors and with concentrations of lipoprotein particles, measured by NMR, are presented. The association of EL mass with categorical variables was examined using Kruskal-Wallis rank test. Data were analyzed in the total group and in men and women separately, because the distributions of CAC, HDL, EL, and many risk factors vary with gender [24,25]. Differences in log-transformed EL mass by gender, and cut-points in waist circumference and other risk factors were measured by t-test. Median CAC scores were compared across quartiles of plasma EL (pre-heparin: 6–268, 268.5–422, 422.5–641, and 641.5–2,043; post-heparin: 102–888, 888.5–1,313, 1,313.5–1,927, and 1,927.5–7,505) using the Wilcoxon test for trend. Ordinal logistic regression is a method appropriate for the analysis of CAC data that has a markedly non-normal distribution and a significant proportion of participants with no detectable CAC [26]. CAC scores were divided into ordered outcome categories (0, 1–10, 11–100, 101–400, and >400) using published criteria [27] as described [26,28]. The association of EL mass with CAC was assessed in multivariable models that also included: (1) gender and age (age and age^2^); (2) established risk factors, gender, and age; and (3) waist circumference, medications (including hormone replacement therapy in women), established risk factors, gender, and age. Established risk factors included total (or LDL) and HDL cholesterol, triglycerides, systolic blood pressure, smoking (current versus never and ex-smokers), race, exercise (none versus any), fasting glucose, and alcohol intake (drinks per week). The results of ordinal logistic regression are presented as the odds ratio (OR) of being in a higher CAC category comparing the highest quartile of EL mass to the lowest quartile. The proportional odds assumption of ordinal regression was satisfied for all models [29]. Data analysis was performed by the authors (KOB, MR, and MW) using Stata 8.1 (Stata, College Station, Texas, United States).

## Results

### Characteristics of Study Participants

The characteristics of the study participants are summarized in Table 1. Compared to the 2002 CDC report on the body weight status of US adults [30], there were fewer individuals of healthy weight and more obese individuals in our cohort. The lipid profiles of our participants were similar to those reported for 20- to 59-year-old participants in the National Health and Nutrition Examination Survey III [31].

**Table 1:**
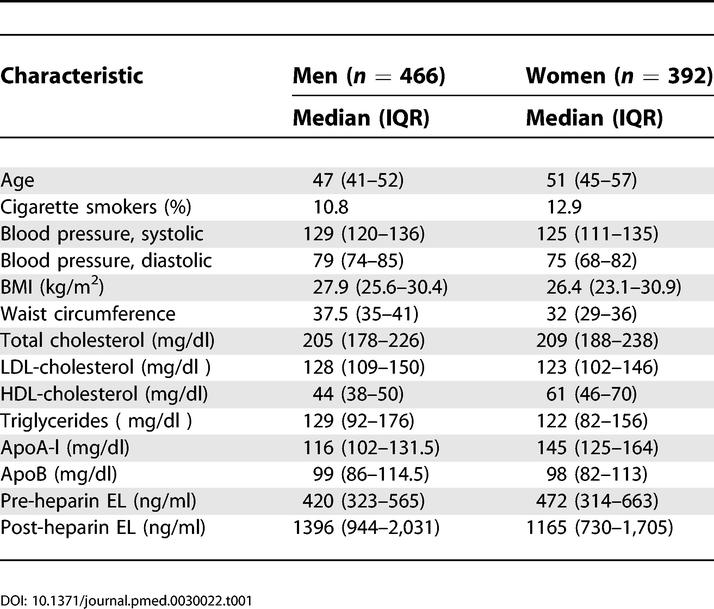
Clinical and Biochemical Characteristics of the Cohort

### Distribution of EL Mass Concentrations in Pre-Heparin and Post-Heparin Plasma

Our previous studies indicated that EL, like HL and LPL [32], is an avid heparin-binding protein (unpublished data), and we suspected that it would be released by injection of heparin in humans. Indeed, a preliminary study in 60 individuals with normal lipid profiles indicated an approximately 3- to 4-fold increase in EL mass concentrations from pre-heparin to post-heparin plasma (unpublished data). Interestingly, EL mass was abundant in pre-heparin plasma of the entire SIRCA cohort (*n* = 858), median = 442 (range 324–617) ng/ml. After administration of heparin, median plasma EL concentrations were approximately 3-fold higher than in pre-heparin samples, 1,313 (888–1,927) ng/ml (*n* = 510). There were no significant differences in pre-heparin EL mass concentrations between men and women. While median post-heparin EL concentrations were 16% lower in women, 1,165 (703–1,705) ng/ml, than men, 1,396 (944–2,031) ng/ml, this difference could be attributed to greater weight in men.

The correlation between pre-heparin and post-heparin EL mass was 0.46, *p* < 0.001. Using linear regression, pre-heparin EL mass accounted for 15% of the variation in post-heparin EL mass. While including age and gender added less than 1% each, waist circumference accounted for 5% of the variation in post-heparin EL mass.

### Association of EL Mass Concentrations with Nonlipid Cardiovascular Risk Factors

Differences in EL mass between groups in the presence and absence of other risk factors were compared by Kruskal-Wallis χ^2^ test. There were significant positive associations between hypertension and both pre-heparin (*p* = 0.004) and post-heparin EL mass (*p* = 0.003). There were significant negative associations between exercise and EL mass in both pre-heparin (*p* < 0.001) and post-heparin (*p* = 0.015) plasma. Smoking was associated with higher post-heparin EL mass (*p* = 0.005).

Correlations of plasma EL mass with age, body mass index (BMI), waist circumference, blood pressure, fasting glucose, and the homeostasis model assessment (HOMA) index, a measure of insulin resistance [33], are shown in Table 2. There was a significant but modest positive correlation between pre-heparin EL mass and age in the entire cohort (*r* = 0.14, *p* = 0.004) and in women (*r* = 0.13, *p* = 0.013) (Table 2). There were significant positive correlations between both pre-heparin (*r* = 0.28, *p* < 0.001) and post-heparin (*r* = 0.22, *p* < 0.001) EL concentrations and both BMI and waist circumference, and these associations remained significant when each gender was examined separately (Table 2). In addition, EL concentrations were greater in obese (BMI > 30) compared to lean men and women in pre-heparin plasma, 575 ± 364 ng/ml versus 456 ± 328 ng/ml, *p* < 0.001, and in post-heparin plasma, 1,820 ± 1,253 ng/ml versus 1,417 ± 836 ng/ml, *p* < 0.001. Both pre- and post-heparin EL mass were correlated with an increased HOMA-IR score: *r* = 0.27, *p* < 0.001 and *r* = 0.24, *p* < 0.001, respectively.

**Table 2:**
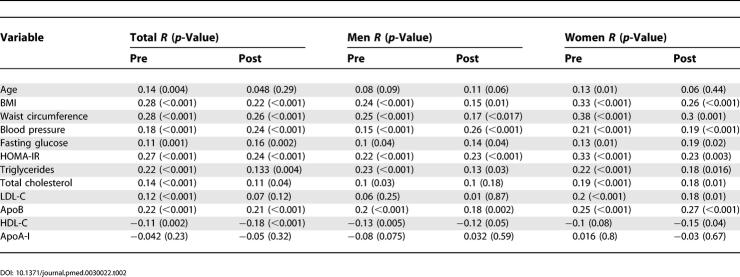
Association of EL Mass with Cardiovascular Risk Factor

### Association of EL Concentrations with Plasma Lipid and Lipoprotein Concentrations

Both pre-heparin and post-heparin EL mass concentrations were positively associated with aspects of an atherogenic lipoprotein profile (Table 2). In pre- and post-heparin plasma, EL mass concentrations were significantly positively correlated with fasting plasma triglyceride and apolipoprotein B (apoB) concentrations in men and women and with LDL-C in women but not men.

To further explore the relationship between plasma EL mass and apoB-containing lipoproteins, we examined the correlations between plasma EL mass and lipoprotein particle size as determined by NMR lipoprotein analysis (Table 3). A significant positive correlation was found between post-heparin EL mass and large VLDL concentrations in men and women, consistent with the positive association of EL with triglycerides. There was a very modest association of EL mass with concentrations of intermediate-size LDL particles in the entire group, which was not significant when assessed separately in men or women. There were no other significant associations of EL mass concentrations with apoB-containing lipoprotein subclasses.

**Table 3:**
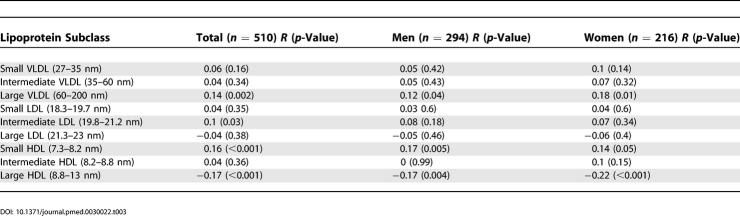
Correlations between Post-Heparin EL Mass and Lipoprotein Subclasses Assessed by NMR

There was a modest, but highly statistically significant, negative association between HDL-C concentrations and both pre- and post-heparin EL concentrations (see Table 2). EL mass concentrations in the lowest and the highest HDL quartile were significantly different, with the difference more pronounced in post-heparin plasma, 1,766 ± 1,231 ng/ml in the lowest HDL quartile versus 1,342 ± 917 ng/ml in the highest (*p* = 0.001). Interestingly, the NMR analysis revealed a negative association of post-heparin EL concentrations with large HDL particles, and a positive association of EL mass with small HDL particles (Table 3). There was no correlation between either pre-heparin or post-heparin EL mass and apoA-I concentrations.

High triglycerides and low HDL-C are two of the criteria used to diagnose metabolic syndrome. We examined the correlation between EL mass and HDL-C concentrations in participants with central obesity as defined by NCEP ATPIII metabolic syndrome waist circumference criteria (greater than 35 inches in women and greater than 40 inches in men). There were stronger negative correlations between post-heparin EL mass in both men and women, *r* = –0.25, *p* = 0.03 and *r* = –0.31, *p* = 0.03, respectively.

**Figure 1:**
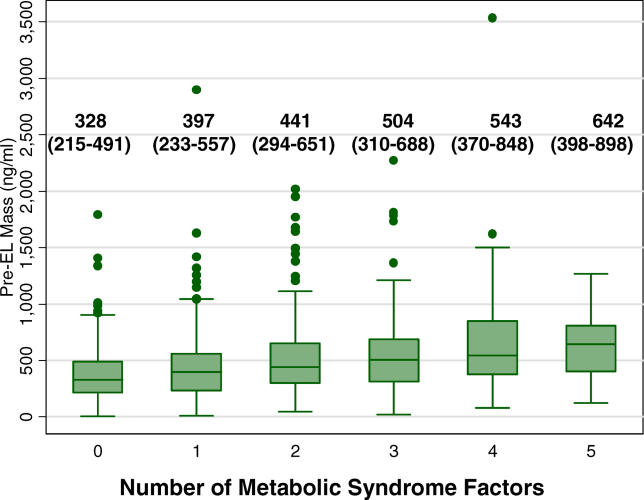
Pre-Heparin EL Mass Concentrations Were Determined in Subgroups According to the Presence of Increasing Numbers of Metabolic Syndrome Factors There was an increase in median pre-heparin EL mass as the number of metabolic syndrome factors increased. Median values and IQR are listed above each box.

Since there were significant correlations between EL mass and each of the metabolic syndrome parameters, we examined differences in median post-heparin EL mass with increasing numbers of metabolic syndrome factors present (Figure 1). There was an additive effect of increasing numbers of metabolic syndrome factors, with median EL mass increasing from 328 ng/ml in individuals with no factors to 642 ng/ml in participants with all five factors present.

### Association of EL Concentrations with Coronary Artery Calcification

**Figure 2:**
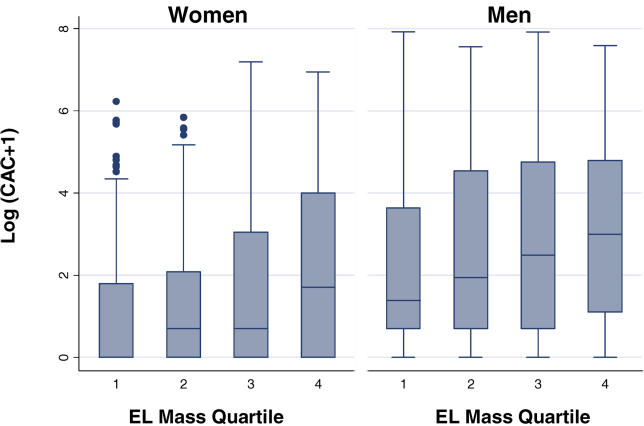
Coronary Artery Calcification Scores Were Compared across Quartiles of Pre-Heparin EL Mass in Both Men and Women The box represents the 25th to 75th percentile, and the line represents the median CAC score within a given pre-heparin EL mass quartile. The whiskers represent the fifth and 95th percentiles. The diamonds are outliers above the 95th percentile. There was an increase in CAC scores as EL mass increased.

CAC scores increased across quartiles of both pre- and post-heparin EL concentrations in men (for trend, *p* < 0.001 for both) and women (for trend, *p* < 0.001 for both) (Figure 2). Using multivariable ordinal regression analyses of the entire cohort, plasma concentrations of both pre- and post-heparin EL mass were significantly associated with CAC after controlling for age, gender, and established risk factors, and after further adjustment for plasma lipids, waist circumference, and vasoactive medications (Table 4). In stratified analyses, the fourth EL quartile compared to the first was associated with higher CAC scores for both pre-heparin EL mass, OR = 1.82 (confidence interval [CI] 1.02–3.24, *p* = 0.043) and post-heparin EL mass, OR = 7.63 (CI 2.44–23.9, *p* < 0.001) in women, while in men pre-heparin EL mass, OR = 1.7 (CI 1.0–2.9, *p* = 0.05) but not post-heparin EL mass, OR = 1.46 (CI 0.7–3.08, *p* = 0.31) was significantly associated with higher CAC scores.

**Table 4:**
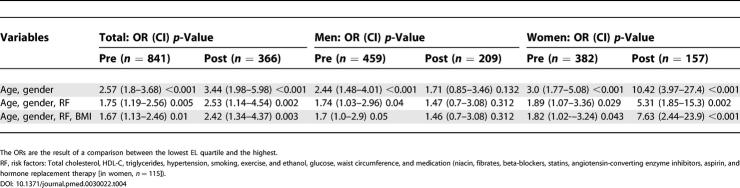
Multivariable Association of Plasma EL Concentrations with Coronary Artery Calcification

## Discussion

We report, to our knowledge for the first time, measurement of plasma concentrations of EL mass in routine, pre-heparin, and post-heparin plasma of human individuals. Using this newly developed sandwich ELISA, we demonstrated that EL mass concentrations in pre-heparin plasma were higher than those of LPL [34–37], and injection of heparin resulted in a 3-fold increase in plasma EL mass to concentrations similar to those reported for HL [34,38]. Importantly, within individuals, the pre-heparin and post-heparin EL concentrations are significantly correlated, suggesting that pre-heparin EL mass may be a surrogate marker for total vascular EL expression. Indeed, most of the associations between post-heparin EL mass and metabolic syndrome factors, cardiovascular risk factors, lipids, and CAC were also found for pre-heparin concentrations. This suggests that future studies of the association of EL mass concentrations with cardiovascular outcomes may be able to be performed using routine, pre-heparin plasma samples.

We previously reported that overexpression of EL in hyperlipidemic mouse models reduced concentrations of apoB-containing lipoproteins [39]. However, in this human study, we found that concentrations of EL are positively associated with plasma concentrations of LDL-C, triglycerides, and apoB, but did not correlate with the number of particles in LDL subfractions. This is in contrast to HL, which hydrolyzes remnant lipoproteins and is negatively associated with remnant particles and positively associated with concentrations of small, dense LDL particles [40]. The positive association of EL with apoB-containing lipoproteins is most likely the reflection of the concomitant increase in VLDL and triglycerides [41] and EL mass found in obese individuals and those with metabolic syndrome.

This is the first study to demonstrate that, in humans, EL plasma concentrations are negatively associated with HDL-C concentrations. While the negative correlations found between plasma EL mass concentrations and HDL-C concentrations are decidedly modest, they are highly statistically significant. The correlation between plasma EL mass and HDL-C may, in fact, underestimate the association of EL activity with HDL-C, given that the EL mass assay may only partially reflect the biological activity of EL. This finding is consistent with published reports in mice that overexpression of EL dramatically reduces HDL-C concentrations [6,9], and either inhibition of EL or genetic deletion causes an increase in HDL-C [9,10,11]. Furthermore, we show that EL concentrations are negatively associated with large HDL but positively associated with small HDL, consistent with the model proven in mice [8], whereby EL hydrolyzes surface phospholipids on large HDL, creating smaller, phospholipid-depleted HDL particles. This pattern of negative correlation with large HDL and positive correlation with small HDL has also been reported for HL [42]. Of significance was the lack of correlation between EL mass concentrations and apoA-I. This finding is consistent with the report by Jahangiri [43] that EL remodeling of HDL to smaller particles does not mediate apoA-I dissociation.

The apolipoprotein content of HDL has been shown to influence the activity of HL and EL toward HDL. Several reports by Patsch et al. [44] and Mowri et al. [45] have suggested that an increase in the apoA-II content increases HL hydrolysis of the triglyceride in the larger HDL_2_ particle. Recent studies by Hedrick et al. [46], using apoA-II transgenic mice, suggest that apoA-II inhibits HL activity, but the addition of apoA-I partially reverses this inhibition. Boucher et al. [47] found similar results in in vitro studies, in which the addition of apoA-II to apoA-I–containing HDL inhibited substrate hydrolysis by increasing the affinity of HL for the HDL particle. The authors reported that the addition of apoA-II to particles increased HDL size and induced a conformational change in apoA-I. In contrast, Caiazza et al. [48] used spherical HDL of identical size, containing cholesteryl ester as the sole lipid core with phosphatidylcholine and apoA-I, apoA-II, or both to determine the influence of apolipoproteins on EL activity. They found that EL hydrolysis of phospholipids in apoA-II–containing HDL was negligible, measurable in apoA-I–containing HDL, and greatest in spherical HDL containing both apoA-I and apoA-II. While the absence of triglycerides in these spherical particles makes application to native HDL difficult, it does suggest that there are differences in the subpopulations of HDL to which HL and EL bind.

Both Jin et al. [49] and Hirata et al. [50] reported that stimulation of cultured endothelial cells with tumor necrosis factor-α and interleukin 1β caused an increase in EL mRNA expression and protein secretion. Elevated plasma concentrations of tumor necrosis factor-α are found in individuals with metabolic syndrome [51]. The positive correlations between plasma EL mass, obesity, and the other metabolic syndrome factors provide indirect evidence of a cytokine-mediated increase in endothelial EL protein secretion in the setting of obesity and metabolic syndrome. The significant difference in EL mass between lean and obese participants also suggests that this may occur in vivo.

An important finding of this study in healthy asymptomatic individuals is that EL mass concentrations are positively associated with CAC, a measure of subclinical atherosclerosis in humans, even after controlling for cardiovascular risk factors, plasma lipids, and vasoactive medications. The observation that EL tends to be a stronger predictor of CAC in women than in men could reflect the fact that HL activity is lower in women [52]. In the presence of less HL activity, EL might have a greater relative influence on HDL metabolism and atherosclerosis.

Importantly, our analysis was based on measurement of EL mass, not EL activity. LPL and HL activity are generally measured in post-heparin plasma by assaying their triglyceride lipase activity. However, EL has much less triglyceride lipase activity compared with these enzymes. Therefore, EL activity in human plasma will need to be assessed based on its phospholipase activity and will require differentiation of EL-specific activity from other sources of phospholipase activity, such as HL. The development of a validated assay will allow determination of the association between EL mass and activity and EL activity with plasma lipids and atherosclerosis.

In summary, EL mass is present in measurable amounts in pre-heparin plasma, and increases 3-fold after administration of heparin. EL concentrations are inversely associated with total HDL-C concentrations and positively associated with obesity, triglycerides, fasting glucose, and hypertension, factors composing the metabolic syndrome. Finally, plasma EL concentrations are associated with subclinical atherosclerosis independent of all established risk factors, including plasma lipids. These data support the concept that plasma EL concentrations may modulate metabolic dyslipidemia and atherosclerosis. Prospective studies will be needed to determine if EL is, indeed, a risk factor.

## Supporting Information

Protocol S1 Detailed Information on the Development and Quality Control of the Sandwich ELISA (40 KB DOC).

**Patient Summary**

**Background**

Atherosclerosis (clogging of the arteries), characterized by progressive injury to blood vessels in critical organs such as the heart and the brain, can lead to heart attacks and strokes. It is the most common cause of death and loss of quality of life in Western societies and is on the rise worldwide. In addition to high blood pressure, diabetes, obesity, and smoking, cholesterol levels are a major risk factor for atherosclerosis. Twenty years ago, we thought that high cholesterol was bad, but now we know that there several types of cholesterol, including “good cholesterol” (called HDL-C) and “bad cholesterol” (LDL-C), and that healthy people have high levels of HDL-C and low levels of LDL-C.

**Why Was This Study Done?**

The researchers who did this study and others had previously found a link between a molecule call endothelial lipase (EL) and atherosclerosis in mice. In mice, EL seems to decrease the levels of HDL-C, the “good cholesterol,” and to make the mice more prone to atherosclerosis. Overall, mice with lower levels of EL seemed to be better off. The question was whether EL levels influenced cholesterol levels and atherosclerosis in humans as well. Human studies until now had searched for a connection between different variants of the EL gene and atherosclerosis, but had not yielded clear answers.

**What Did the Researchers Do and Find?**

In this study, the researchers directly measured levels of EL in the blood of over 800 human participants and looked for links with cholesterol levels and other atherosclerosis risk factors. They found that, just as in mice, higher blood concentrations of EL are linked with lower levels of HDL-C (the effect was clear but not very large). They also found that higher concentrations of EL were linked with other risk factors for atherosclerosis such as obesity and hypertension. Finally, they found that raised EL concentrations were linked with signs of early atherosclerosis in the participants, and this link held true even when they corrected for all other risk factors, i.e., when they compared people who had the same weight, blood pressure, and cholesterol levels.

**What Does This Mean?**

The study found a link in humans between high EL concentrations in the blood, low HDL-C levels, and early stages of atherosclerosis. This suggests (but does not prove) that EL concentrations influence the risk of atherosclerosis and could be used to predict an individual's risk. Proving that EL levels predict risk can only be done in so-called “prospective studies” (studies that look forward over time, rather than go backward). Such studies would measure EL blood concentrations in a large number of people now, subdivide them into groups that differed in their EL levels but not in any other factors known to influence atherosclerosis, and then check years later whether there are differences in atherosclerosis, heart attacks, and strokes between the groups. Such studies will take time. While they are ongoing, it seems worth exploring the possibility that treatments to reduce EL levels might help prevent atherosclerosis.

**Where Can I Find More Information Online?**

Patient information pages from the National Lipid Association: http://www.lipid.org/clinical/patients/

Medline Plus pages on vascular diseases: http://www.nlm.nih.gov/medlineplus/vasculardiseases.html

A collection of atherosclerosis resources for patients and families from the Guam Medical Library:

http://guam-dl.slis.ua.edu/patientinfo/cardiology/cardiovascular/atherosclerosis.html

Atherosclerosis page at the University of Maryland Web site: http://www.umm.edu/cardiac/athero.htm

American Heart Association Web site:

http://www.americanheart.org

## Abbreviations

BMI: body mass index
CAC: coronary artery calcification
CAD: coronary artery disease
CI: confidence interval
EL: endothelial lipase
HDL-C: high-density lipoprotein cholesterol
HL: hepatic lipase
HOMA: homeostasis model assessment
IQR: interquartile range
LDL: low-density lipoprotein
LPL: lipoprotein lipase
NMR: nuclear magnetic resonance
SIRCA: Study of the Inherited Risk of Atherosclerosis
VLDL: very low-density lipoprotein
