# Supplementary material for: Endothelial Lipase Concentrations Are Increased in Metabolic Syndrome and Associated with Coronary Atherosclerosis
Source: PLoS Med. 2005 Dec 20;3(2):e22. doi: 10.1371/journal.pmed.0030022 (PMC1316064; doi:10.1371/journal.pmed.0030022)
Supplement: Supplementary file 1 [file pmed.0030022.sd001.doc]

**Supporting Information**

*Materials –* Yeast nitrogen base, avidin/4’-hydroxyazo-benzene-2-carboxylic acid (HABA), ο-phenylene diamine (OPD), streptavidin-HRP conjugate, and bovine serum albumin were from Sigma-Aldrich Co. (St. Louis, MO). PICZα, DH5α competent E. coli, and Zeocin were from Invitrogen (Carlsbad, CA). Tryptone, peptone, yeast extract and agar were from Becton Dickinson (Sparks, MD). Nunc immunoplates were through Fisher Scientific (Fairlawn, NJ). All other reagents were best quality commercially available.

*Biotin-labeling of anti-human EL antibody –* A polyclonal antibody to EL was produced by intravenous injection of a recombinant adenovirus coding for the human EL transgene into rabbits (53). Presence of antibody was determined by western blot with serum from immunized rabbits at weekly intervals until the titer had maximized. Rabbit anti-human endothelial lipase IgG was purified from antiserum using a Protein G sepharose HiTrap column on an Äkta FPLC system (Amersham Pharmacia Biotech, Uppsala, Sweden).

A portion of this IgG was labeled with biotin using an EZlink Sulfo-NHS-LC biotinylation kit according to the manufacturer’s instructions (Pierce, Rockford, IL). Biotin incorporation was calculated by measuring the absorbance at 500 nm of 0.9 ml avidin/HABA solution (6 µM avidin, 0.3 mM HABA) (A1) then determining the change in absorbance after addition of 0.1 ml biotin-labeled antibody (A2). The percent incorporation is then calculated: D_A500_ = 0.9 (A1-A2)

Biotin (M) = D_A500/34000_

Antibody (M) = [antibody in mg/ml] x 0.1/MW

Molar degree of biotin incorporation = [biotin]/[antibody]

Biotin incorporation was determined to be 2 moles biotin/ mole antibody

*Expression of recombinant human EL in Pichia pastoris –* Polymerase chain reaction was run using a pcDNA 3.0-EL construct and the following primers:

5’: TCTCTCGAGAAAAGAGAGGCTAGCCCCGTACCT

3’: TTGTTCTAGAAAGTTTATCATCATCATCTGGAAGCTCAAC

Both the EL cDNA and the PICZα *Pichia pastoris* vector were cut with XhoI and XbaI, then ligated with T4 DNA ligase. The EL-containing vector was transformed into DH5α. Positive clones were selected from Zeocin-containing (50 µg/ml) low salt LB agar (10 g tryptone, 5 g NaCl, 5 g yeast extract), expanded in low salt LB medium with Zeocin, and the vector purified by spin column (Qiagen, Valencia, CA) The PICZα-EL vector was cut with Sac I, then transformed into X33 *Pichia pastoris* according to the manufacturer’s instructions*.* The transformed P. pastoris was plated at different dilutions onto yeast extract-peptone-dextrose (YPD) agar with 100 µg/ml Zeocin, positive colonies picked and expanded in 25 ml Zeocin-containing BMG (100 mM potassium phosphate, pH 6.0, 1.34% yeast nitrogen base, 0.00004% biotin, 1% glycerol) medium in a 250 ml baffled flask at 30^0^C, 280 rpm overnight. The yeast suspension was diluted to OD_600nm_ =1 and used to innoculate 1 liter of BMM medium (100 mM potassium phosphate, pH 6.0, 1.34% yeast nitrogen base, 0.00004% biotin, 0.5% methanol) in a 4 L baffled flask. This culture was grown at 30^0^C, 280 rpm, increasing the methanol concentration by 0.5% every 24 H, to a maximum of 2%. The presence of EL in the medium was tested by western blot.

*Purification of recombinant EL –* Yeast culture medium containing secreted EL was adjusted to pH 7.4 using phosphate buffer, pH 8.0. The medium was applied to a c-myc antibody agarose column by peristatic pump at 4^0^C. After washing with 20 column volumes of phosphate-buffered saline, the column was eluted with 0.1 M glycine, pH 4.0.

Peak fractions were detected by A_280_ absorbance, combined and dialyzed against PBS overnight at 4^0^C. Protein was quantified by bicinchoninic acid assay (Pierce, Rockford, IL). Purity was assessed by to be >95% SDS-PAGE and silver stain. This protein was used as the standard for determination of plasma EL mass by sandwich ELISA. The reactivity of the purified protein with the polyclonal anti-EL antibody was confirmed by western blot and by use of the protein in standard curves by ELISA.

*Sandwich enzyme-linked immunosorbent assay of EL -* The wells of a 96-well microtiter plate were coated with 10 μg/ml rabbit anti-human EL antibody diluted in carbonate-bicarbonate buffer, pH9.2. The wells were blocked with 3% BSA in phosphate-buffered saline (PBS). After washing 3 times with PBS, 0.1% Tween20, 100 μl of various concentrations of purified recombinant human EL in PBS, 1% BSA, were added to the wells as a standard. Plasma samples were diluted 1:10 in PBS, 100 μl volume, and applied to the wells. After washing 3 times, as described above, specifically bound protein was incubated with 100 μl/well of a 5 μg/ml solution of biotin-conjugated rabbit anti-human EL antibody in PBS, 1% BSA. After washing, 100 μl of a 1 μg/ml solution of streptavidin-horseradish peroxidase conjugate in PBS, 1% BSA, was incubated for 1 hour. After washing 5 times, specifically bound protein was detected with 100 μl/well of a 417 ug/ml solution of o-phenylenediamine. The reaction was stopped with 100 μl/well of 2.5M sulfuric acid and the plate read at 490 nm. A standard curve of 490 nm absorbance versus the known concentrations of EL was constructed. The concentration of the plasma samples was determined by comparison to the standard curve multiplied by the dilution factor of ten.

*Quality control of the endothelial lipase enzyme-linked immunosorbent assay*

*Sensitivity -* The accuracy with which this method measures EL mass was determined by assaying pooled plasma, diluted 1:10 in PBS, with and without the addition of known amounts of recombinant EL protein: 2ng, 18 ng, and 25 ng. The results of this assay measured approximately 75% of the added 2 ng, 80% of the added 18 ng and 90% of the added 25 ng. The assay is, therefore, sensitive enough to discern small differences in EL mass.

Pooled post-heparin plasma was also diluted 1:100, 1:50, 1:25 and 1:10. The mass obtained at the four dilutions was 281±27 ng/ml with only the 1:100 dilution tending to over-estimate the concentration. All plasma samples used in these studies were diluted 1:10 in PBS.

*Specificity –* The cross-reactivity of the polyclonal EL antibody to both rHL and rLPL was tested a minimum of 5 times on Western blot without evidence of cross-reactivity.

The specificity of the capture of EL to the coating antibody was determined by a solution competition assay. Pooled post-heparin human plasma was diluted 1:10 in the presence of 100µg/ml rabbit anti-EL antibody, and incubated for 10 minutes before application to wells coated with the same antibody. Greater than 95% of the absorbance, relative to pooled plasma in the absence of solution-phase antibody, was eliminated. Specificity was also determined by 3 repetitions of an assay determining rabbit anti-EL antibody capture of rHL and rLPL in culture medium, each diluted 1:10. The absorbance of rLPL was <5% above background and rHL was <10% above background.

A mixing assay was run with a fixed concentration of EL, 125 ng/ml, and approximate concentrations (based on activity) of both rHL and rLPL added, up to 500 ng/ml. At the highest concentrations of either rHL or rLPL, the calculated concentration of EL was 140 ng/ml and 121 ng/ml, respectively.

An ELISA was performed with pre-heparin plasma from a compound heterozygote, A174T and T383M, subject with a HL plasma level of 14 ng/ml (54). The mass of EL in this HL deficient plasma was found to be 422 ng/ml; very consistent with the mean mass in the study population.

*Sample handling -* To examine the effect of sample handling on measurement of EL mass, comparisons were made of EL mass measured from the same samples, either fresh or frozen. Blood was drawn from 13 individuals into 1 mM EDTA-containing tubes and centrifuged at 3000 rpm for ten minutes to separate the plasma. An aliquot of plasma was diluted 1:10 in PBS and assayed for EL mass. A second aliquot was frozen in a cryotube and placed in a -80^0^C freezer under the conditions used to store the plasma samples from the SIRCA cohort. The mass in the fresh samples, 737± 276 ng/ml, compared favorably to the mass measured on the frozen samples, 803 ± 272 ng/ml. All samples measured in this study were held at -80^0^C until assayed. The correlation between fresh and frozen samples was 0.98, p<0.001. There should, therefore, be no variability introduced based on differences in sample handling.

*Reproducibility*

Standard curves were run ten times with recombinant EL protein and the polyclonal rabbit anti-human endothelial lipase antibody. The slope of this curve was 0.026±0.008.

To assess the reproducibility of the ELISA for human endothelial lipase, pooled plasma from six donors was separated into 100 µl aliquots and frozen at -80^0^C until use. This plasma was diluted 1:10 in PBS and assayed. The mass determined in 20 different assays was 863±81 ng/ml with a range of 775-981 ng/ml, or 11.2-14.4 nM. The intra-assay variability was 6.8% and the inter-assay variability was 10.3%. Both values are within the acceptable range for industrial quality control. The assay, therefore, shows a high degree of reproducibility.

*Effect of	Potentially Interfering Substances*

To determine if elevated levels of metabolites could interfere with the detection of EL in human plasma, concentrations of bilirubin (100 μM), uric acid (150 μg/ml) and creatinine (100 ng/ml) ten-fold higher than the normal range were added to plasma samples and compared to untreated plasma. Bilirubin, uric acid and creatinine produced minimal interference, +/- 10% of untreated plasma.
